# Challenges in posterior uveitis—tips and tricks for the retina specialist

**DOI:** 10.1186/s12348-023-00342-5

**Published:** 2023-08-17

**Authors:** Manuel Paez-Escamilla, Sonny Caplash, Gagan Kalra, Jamie Odden, Danielle Price, Oscar C. Marroquin, Stephen Koscumb, Patrick Commiskey, Chad Indermill, Jerome Finkelstein, Anna G. Gushchin, Andreea Coca, Thomas R. Friberg, Andrew W. Eller, Denise S. Gallagher, Jean C. Harwick, Evan L. Waxman, Jay Chhablani, Gabrielle Bonhomme, Colin Prensky, Alexander J. Anetakis, Joseph N. Martel, Erika Massicotte, Raphaelle Ores, Jean-Francois Girmens, Thomas M Pearce, Jose-Alain Sahel, Kunal Dansingani, Mark Westcott, Marie-Helene Errera

**Affiliations:** 1 Department of Ophthalmology, University of Pittsburgh School of Medicine, Pittsburgh, PA USA; 2 Clinical Analytics, University of Pittsburgh, Pittsburgh, PA USA; 3 Department of Rheumatology, University of Pittsburgh School of Medicine, Pittsburgh, PA USA; 4 Department of Ophthalmology, McGill University Campus Outaouais, Gatineau, QC Canada; 5 Centre hospitalier National des Quinze-Vingts, Paris, France; 6 Division of Neuropathology, University of Pittsburgh Medical Center, Pittsburgh, PA USA; 7 Department of Uveitis, Moorfields Eye Hospital, NHS Foundation Trust, London, UK; 8 UPMC Eye Center, University of Pittsburgh School of Medicine, 203 Lothrop Street, Pittsburgh, PA 15213 USA

**Keywords:** Birdshot choroidopathy, Check-point inhibitors retinopathy, Masquerades, MEK inhibitors retinopathy, Multifocal choroiditis uveitis, Ocular inflammation, Posterior uveitis, Punctate inner choroidopathy, Serpiginous choroiditis, Vogt- Koyanagi-Harada, White dot syndromes

## Abstract

**Purpose:**

Posterior uveitis is a common chorioretinal pathology affecting all ages worldwide and is a frequent reason for referral to the retina clinic. The spectrum of etiologies for uveitis is very broad and includes infectious and auto-immune diseases. Inflammation can be confined to the eye or may be a part of systemic disease. A useful outline is therefore proposed to aid in the correct diagnosis of these challenging entities. The situation is further complicated by the fact that many neoplastic conditions resemble features of posterior uveitis; they are known as “masqueraders of uveitis”. Here, we summarize different posterior uveitides that present with rare findings, along with masqueraders that can be difficult to distinguish. These conditions pose a diagnostic dilemma resulting in delay in treatment because of diagnostic uncertainty.

**Methods:**

An extensive literature search was performed on the MEDLINE/PUBMED, EBSCO and Cochrane CENTRAL databases from January 1985 to January 2022 for original studies and reviews of predetermined diagnoses that include posterior uveitic entities, panuveitis and masquerade syndromes.

**Results:**

We described conditions that can present as mimickers of posterior uveitis (i.e., immune check-points inhibitors and Vogt-Koyanagi-Harada-like uveitis; leukemia and lymphoma associated posterior uveitis), inflammatory conditions that present as mimickers of retinal diseases (i.e., Purtscher-like retinopathy as a presentation of systemic lupus erythematosus; central serous chorioretinopathy masquerading inflammatory exudative retinal detachment), and uveitic conditions with rare and diagnostically challenging etiologies (i.e., paradoxical inflammatory effects of anti-TNF-α; post vaccination uveitis; ocular inflammation after intravitreal injection of antiangiogenic drugs).

**Conclusion:**

This review of unique posterior uveitis cases highlights the overlapping features of posterior uveitis (paradoxical inflammatory effects of anti -TNF α and uveitis; Purtscher-like retinopathy as a presentation of systemic lupus erythematosus, …) and the nature of retinal conditions (ischemic ocular syndrome, or central retinal vein occlusion, amyloidosis, inherited conditions like retinitis pigmentosa, autosomal dominant neovascular inflammatory vitreoretinopathy (ADNIV), etc.…) that may mimic them is represented. Careful review of past uveitis history, current medications and recent vaccinations, detailed examination of signs of past or present inflammation, eventually genetic testing and/ or multimodal retinal imaging (like fluorescein angiography, EDI-OCT, OCT-angiography for lupus Purtscher-like retinopathy evaluation, or ICG for central serous retinopathy, or retinal amyloid angiopathy) may aid in correct diagnosis.

**Supplementary Information:**

The online version contains supplementary material available at 10.1186/s12348-023-00342-5.

## Introduction

Ocular inflammatory disease is a leading cause of vision loss worldwide. In the United States, uveitis has a reported incidence of 52.4/100 000 person-years [1]. Uveitis encompasses a wide spectrum of diseases that can affect virtually every part of the eye. Inflammation can be confined to the eye or may be part of systemic disease. Thus, ocular inflammation possesses a significant challenge as many diseases may present as posterior uveitis masqueraders, mimickers of retinal diseases or inflammatory diseases with obscure, rare etiologies. When using the term “Uveitis Masqueraders”, most of these are simply different conditions with similar phenotypes or clinical appearance.

Uveitis masquerade syndromes represent as many as 5% of patients seen in uveitis clinics and the frequency of neoplastic masquerade syndrome is usually 2.5% of patients [2].

Oncologic conditions such as lymphoma, hematologic malignancy, and paraneoplastic syndrome can present with ophthalmic manifestations, mimicking uveitis [2–7]. Infectious conditions, like Lyme disease, tuberculosis, syphilis, herpes and zoster virus have diverse ocular presentations, and treatment modalities. Patients who are immuno-compromised, either as a result of HIV infection, cancer, or the treatment thereof, are at increased risk of infectious uveitis with atypical presentations. Additionally, ocular syphilis and toxoplasmosis may be misdiagnosed for herpetic acute retinal necrosis.

The growing field of drug-related uveitis should also be considered. Vaccines, working primarily to stimulate an immune response against a target infection, can rarely and inadvertently cause uveitis, which may have implications for treatment and subsequent personal vaccine management [8–15].

Ischemic vascular conditions can often present with concomitant inflammation reminiscent of uveitis [16, 17]. It is also important to consider drug-related uveitis when discussing masquerading presentations [18, 19]. The correct diagnosis often requires a comprehensive rheumatologic and ophthalmological evaluation including: clinical history, multimodal imaging and systemic workup.

The purpose of this article is to review some of these conditions, including clinical features that aid in identification and distinct findings on multi-modal imaging. The present study attempts to concentrate knowledge of ocular conditions that might mimic intraocular inflammation diseases.

We reviewed the most common conditions that present as mimickers of posterior uveitis and conversely, inflammatory conditions that present as mimickers of retinal diseases, and uveitis conditions with rare and diagnostically challenging etiologies.

Their typical and atypical presentations are reviewed as outlined below. Diagnostic strategies among the entities described below are also presented and discussed.

1. Common drug related, retinal vascular, neoplastic and other conditions that may present as uveitis masqueraders

a Drug related:
  - Immune check-points inhibitors (ICI) and Vogt-Koyanagi-Harada-like uveitis
  - Mitogen-activated protein kinase enzymes (MEK) inhibitor-associated retinopathy
  - Post vaccination uveitis: mRNA COVID-19 vaccinations, Bacille Calmette-Guerin (BCG) vaccine, tuberculin skin testing, measles, mumps, and rubella, influenza vaccination, hepatitis B, human papillomavirus, and varicella vaccines.
  - others: intravitreal injection of antiangiogenic drugs, diethylcarbamazine
b Neoplastic conditions: Leukemia and Lymphoma associated Posterior Uveitis
c Retinal vascular diseases: Giant cell arteritis (GCA), ischemic ocular syndrome, central retinal vein occlusion (CRVO)
d Idiopathic eye-limited disorder not conforming to a defined syndrome:
  1. Central Serous Chorioretinopathy (CSCR) masquerading inflammatory exudative retinal detachment
  2. Rhegmatogenous retinal detachment (RD) masquerading as exudativepanuveitis with anterior chamber inflammatory reaction
  3. Hereditary retinal disease
  4. Myopic degeneration and drusen
e Systemic disorders
  1. Purtscher-like retinopathy as a presentation of systemic lupus erythematosus (SLE)
  2. Amyloidosis

2. Misdiagnoses among Uveitis entities

a Infectious
  - Acute Retinal Necrosis (ARN) misdiagnoses
    - Syphilis (sectoral retinitis)
    - Retinochoroiditis secondary to Toxoplasma gondii
  - Presumed Tuberculous Serpiginous-Like Choroiditis (SLC) and Multifocal Serpiginoid Choroiditis masquerading Serpiginous Choroiditis (SC)
  - COVID
b Non-infectious
  1. White dot syndromes misdiagnoses
    b.1.1. Acute Posterior Multifocal Placoid Pigment Epitheliopathy (APMPPE) misdiagnosed for VKH
    b.1.2 Other mimickers for Vogt-Koyanagi-Harada (VKH) disease
    b.1.3. Choroidal granulomas in sarcoidosis masquerading like birdshot choroidopathy (BC) lesions on ICG
    b.1.4. Presentations of birdshot (BC) with minimal or absent birdshot spots
c Others
  1. Extensive scarring throughout the fundus: progressive subretinal fibrosis and uveitis syndrome (PSFU).
  2. Sarcoid Choroidal Granulomas presenting as Paving Stone lesions
  3. Drug related: Paradoxical inflammatory effects of anti-Tumor necrosis factor α (TNFα) and uveitis

## Methods

The study was conducted according to the Preferred Reporting Items of Systematic Reviews (PRISMA) guidelines [20].

A literature search and subsequent screening of articles was conducted in December 2021 by three authors (MPE, SC and MHE). PubMed served as the primary database for the electronic literature search, although EBSCO and Cochrane were also surveyed. We systematically reviewed the available literature on neoplastic and nonneoplastic inflammatory masquerade syndromes. Literature searches were performed using electronic medical databases of the following keywords: masquerade syndromes, inflammatory ocular diseases and uveitis. The search timeframe was not limited by a specific date, but rather by the results of the articles retrieved.

The retrieved articles were initially screened by title and abstract, and articles with the relevant titles were then screened by full text using predefined inclusion and exclusion criteria. Inclusion criteria included 1) the paper must be written in or available in English and 2) the paper discussed the presentation and management of masquerade syndromes, inflammatory and infectious ocular diseases and uveitis. Exclusion criteria included 1) papers involving patients only with other inflammatory ocular diseases (episcleritis, scleritis) and anterior uveitis, intermediate uveitis. This article specifically excluded entities such as AZOOR, and autoimmune retinopathy for the reason that typically patients with this condition do not present with sign of overt intraocular inflammation; 2) the paper did not clearly diagnose the patient with a masquerade syndrome.

3) citations were from grey literature. The full article was screened in cases where the relevance was unclear from the abstract. Relevant articles were ultimately compiled into a database and removed of duplicates (Supplemental table).

No research ethics approval was needed for this study, as there were no human or animal participants included. The study protocol complied with the tenets of the Declaration of Helsinki.

## Results

A total of 535 studies were identified in the literature search. After screening study titles and abstracts and excluding duplicates, 148 articles were excluded and 387 articles remained eligible for full-text examination. Of these, 230 met all inclusion criteria (Supplemental figure). Specific diagnoses selected to be included in the study can be found in the Supplemental table.

**Fig. 1:**
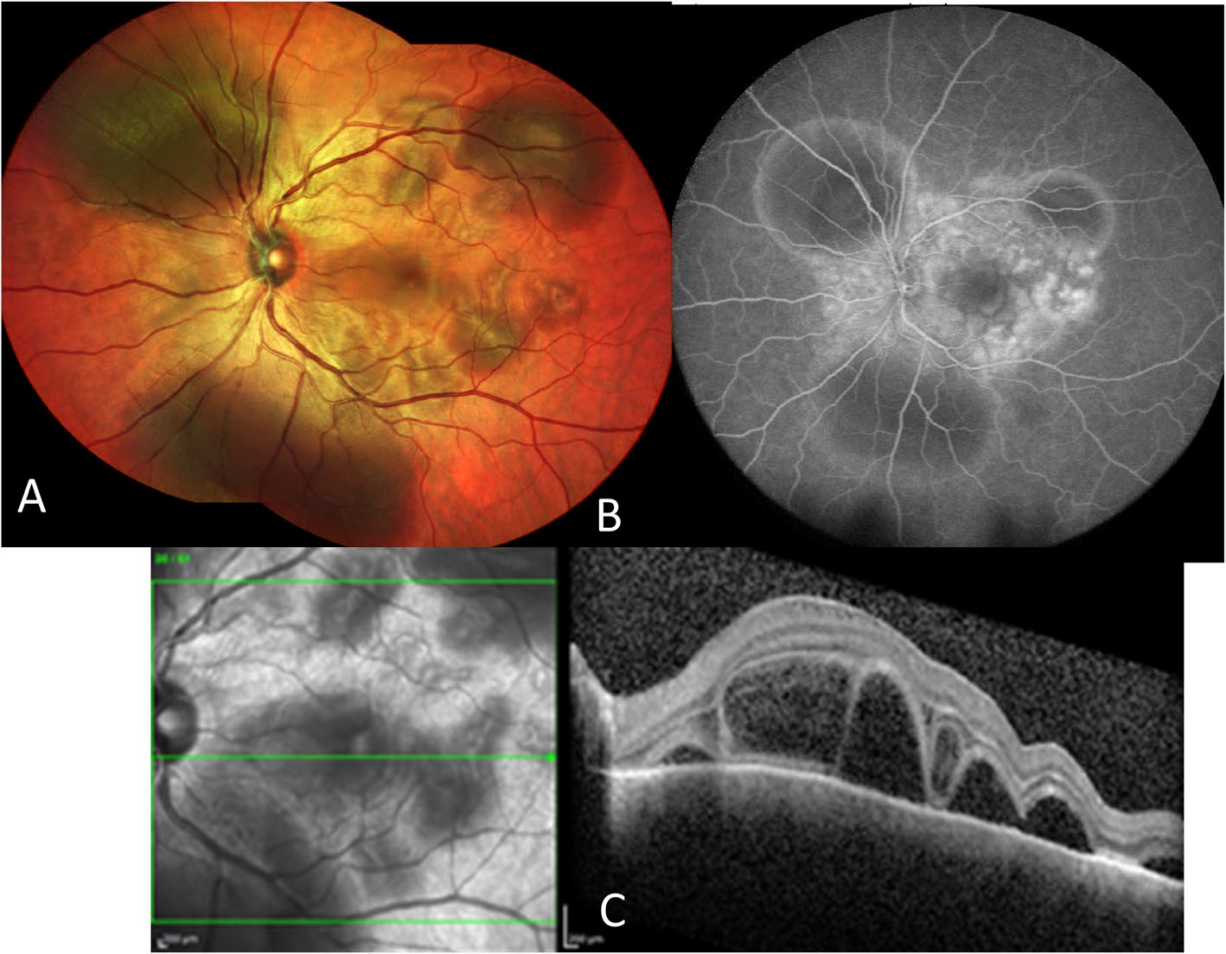
Multimodal imaging in a 51-year-old female with acute pseudo-Vogt-Koyanagi-Harada (VKH) following one month treatment with a combination of ipilimumab/nivolumab (CTLA-4 and PD-1 checkpoint blockade inhibitors) for a malignant melanoma of the skin. **A** The fundus photograph of the left eyes reveals multiple deep yellow choroidal lesions with areas of the subretinal detachments in the peripapillary region. **B** Note multiple hyperfluorescent retinal pigment epithelium leaks on fluorescein angiography with hyperfluorescent dye pooling beneath subretinal fluid. **C** Optical coherence tomography (OCT) in pseudo-VKH showed a multilobular serous macular detachments, with subretinal hyper-reflective material within the subretinal fluid that likely represents fibrin and a part of the outer segment layer
    Herein, we highlight the VKH-like uveitis (Fig. 1) that has been frequently reported in association with checkpoint inhibitors^.^. To date, 126 cases of anterior uveitis, intermediate uveitis, posterior uveitis, and panuveitis have been reported in the literature [24]. Patients have been reported to typically develop intraocular inflammation at a median of 9 weeks after initiation of ICI, while 83.6% of patients develop uveitis within 6 months [24].
    Furthermore, in melanoma, the development of VKH**-**like uveitis and skin-related toxicities, such as rash and vitiligo, secondary to immune checkpoint inhibitors correlates with increased tumor response and prolonged survival*.* In those patients, malignant melanoma cells and normal choroidal melanocytes share a common target epitope for T-cell recognition. Therefore, the release and activation of T‐cells from PD‐1 inhibition and the CTLA-4 pathway would lead to T cells targeting of both malignant melanocytes and non-malignant choroidal melanocytes [25]. Usually, cessation of ICI use is discussed with the oncologist taking into account the risk/benefit ratio. Treatment with topical or systemic corticosteroid therapy is often associated with improvement in symptoms and disease rarely recurs [26–28].
    - Mitogen-activated protein kinase inhibitors (MEKi) associated retinopathy.
    Mitogen-activated protein kinase inhibitors MEKi are now largely used for treatment of advanced melanoma in combination with B-rab enzyme inhibitors (BRAFi) such as vemurafenib and dabrafenib. MEKi have been associated with a wide phenotype of retinal damage with an incidence that ranges from 5 to 75% [29–31]. The incidence of retinopathy has been reported at its highest in the first two cycles [32]. The incidence of retinopathy is also drug-specific, with higher incidence (57%) reported in patients who receive vemurafenib combined with cobimetinib. [32, 33]. Patients often present with symptoms of photosensitivity, blurred vision and reduced vision [32]. Clinical presentation ranges from mild, with small multifocal and bilateral subretinal detachments [34], to severe, with intraretinal fluid or cysts, and a disarrangement of the outer retinal layers (see Fig. 2) [35–37]. The mechanism behind BRAF/MEK inhibitor induced panuveitis, which clinically closely resembles the VKH disease, might be related to its interference with the MAPK pathway, which is involved in the T-cell receptor signaling pathway. In the VKH disease pathogenesis, CD4 + and CD8 + cells (T cells) target melanocytic antigens in the choroid and RPE, which impair the outer blood retinal barrier [38].

1. Common drug related, retinal vascular, neoplastic and other conditions that may present as uveitis masqueraders
  a Drug related
    - Immune check-points inhibitors (ICI) and Vogt-Koyanagi-Harada (VKH)-like uveitis.
    Anti-PD-1 (programmed cell death protein 1)/PD-L1 (programmed death-ligand 1) and anti-CTLA-4 (cytotoxic T-lymphocyte associated protein 4) immunotherapy has revolutionized the treatment of metastatic non-small cell lung cancers, and melanomas, with a significant improvement in survival observed in some patients [21, 22]. Currently, six immune check-points inhibitors (ICI)s have been approved by the US FDA. New ocular and orbital side-effects have been reported in less than 1% of patients following CTLA-4 and PD-1/ PD-L1 checkpoint blockade inhibitors (nivolumab, ipilimumab, and pembrozulimab). These include peripheral ulcerative keratitis, uveitis (anterior uveitis, panuveitis, posterior uveitis, or optic neuritis), choroidal neovascularization, melanoma-associated retinopathy, thyroid-associated orbitopathy and idiopathic orbital inflammation [23].
    The standard differential diagnoses for serous retinal detachment are as follows: Vogt-Koyanagi-Harada (VKH) disease, posterior scleritis, acute posterior multifocal placoid pigment epitheliopathy (APMPPE), uveal effusion syndrome, lupus choroidopathy, sarcoidosis, sympathetic ophthalmia, leukemia, metastatic carcinoma, uveal lymphoid infiltration, drug induced uveitis, central serous chorioretinopathy, and exudative retinal detachments due to malignant hypertension. Nonetheless, an often-overlooked etiology in cases of bilateral VKH-like uveitis is treatment with ICI (Fig. 1).

**Fig. 2:**
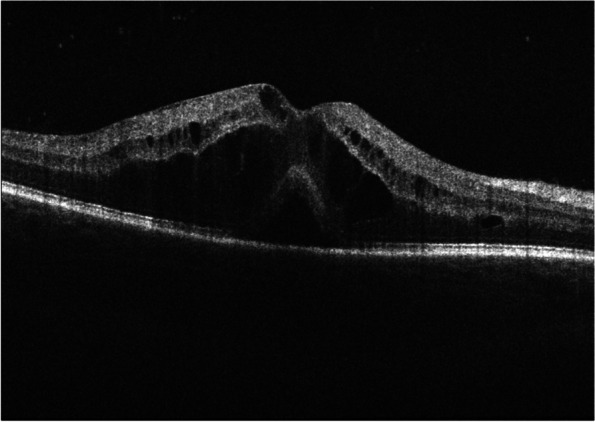
OCT showing a cystoid macular edema and a serous neuroretinal detachment of the fovea in a 39-year-old male with widespread metastatic melanoma, stage IV disease, treated by BRAF/MEK inhibitor therapy (ecorafenib/binimetinib) since the last 3 months
    Ocular adverse effects impact dosing of MEKi. The United States Prescribing Information for trametinib and cobimetinib recommends: “an ophthalmological examination at regular intervals during therapy and at any time a new or worsening visual disturbance is reported. In the case of a grade 2–3 retinal pigment epithelial (RPE) detachment, trametinib should be withheld without change of the dose of dabrafenib and restarted at a lower dose if resolution or improvement is documented within three weeks. If no improvement after three weeks, recurrence, or retinal vein occlusion (RVO), the drug should be discontinued” [39]. For cobimetinib, therapy should be withheld for serous retinopathy until visual symptoms improve with resumption at a lower dose only if symptoms improve over four weeks. For recurrent symptoms or any RVO, the drug should be discontinued permanently [39]. MAPK pathway inhibition can rarely lead to severe panuveitis, which tends to resolve within months with treatment discontinuation and/or treatment with corticosteroids either systemic or local [35].
    Relatedly, inhibitors of fibroblast growth factor receptor (FGFRi) such as ponatinib, dovitinib, erdafitinib, and pemigatinib have a similar presentation of ocular adverse effects. FGFRi are used in the treatment of advanced urothelial cancers with certain FGFR mutations, and previously treated advanced cholangiocarcinomas with FGFR2 gene alterations. These drugs are also associated with a similar type of serous retinopathy to that seen with the MEKi, possibly because the FGFR pathway intersects with the MEK pathway [29, 36].
    - Post vaccination uveitis: mRNA COVID-19 vaccination, Bacille Calmette-Guerin (BCG) vaccine, tuberculin skin testing, measles, mumps, and rubella, influenza, hepatitis B, human papillomavirus, and varicella vaccines.
    There have been numerous published cases of anterior, episcleritis, scleritis, and posterior/ panuveitis after mRNA COVID-19 vaccinations [37]. According to the US Food and Drug Administration Vaccine Adverse Event Reporting System, 851 cases of uveitis after COVID-19 vaccination involved mRNA or adenovirus vector vaccines from the reports processed as of 06/24/2022 [40]. A large multinational case series including 70 patients has shown that the most common events were anterior uveitis (58.6%), followed by posterior uveitis (12.9%) and scleritis (10.0%). The mean time to event was 5 days and 6 days (range, 1–14 days) after the first and second dose of vaccine, respectively. Among all patients, 36 (54.1%) had a previous history of ocular inflammatory event [41]. Clinical presentation can vary widely with reported cases of: new onset of panuveitis in Behçet’s disease, multiple evanescent white dot syndrome (MEWDS), APMPEE, ampiginous choroiditis, and an exacerbation of VKH [41–47]. There have also been reports of reactivated underlying infectious disease, specifically herpetic uveitis, keratitis, and acute retinal necrosis (ARN) as well as toxoplasma retinochoroiditis and tubercular choroiditis after COVID-19 vaccines [42, 48–52].
    Isolated case reports and series have implicated nearly all commercially available vaccines, including: Oxford-AstraZeneca vaccine (ChAdOx1 nCoV-19), ModernaTX vaccination (mRNA-1273), and Janssen Johnson & Johnson vaccine (Ad26.COV2), Sinotec and Covi-shield [14, 15, 42, 53, 54]. Despite this, there is limited large-scale data on the incidence of uveitis among specific vaccines. One study investigated the incidence of uveitis after administration of the BNT162b2 mRNA SARS-CoV-2 vaccination finding that of the 23 eyes studies, 21 presented with anterior uveitis while the remaining 2 presented with MEWDS. The authors suggest that the anterior uveitis was triggered by increased reactogenicity to the second dose of the vaccine, as none of the affected patients suffered from uveitis after the first dose [14]. A population-based study of over two million patients investigating the incidence of uveitis after the BNT126b2 vaccine estimated an age-gender adjusted standardized incidence ratios (SIR) of uveitis after the first dose of 1.41 (95% CI, 1.15–1.71) along with a 21-day attributable risk of 1.12 cases per 100,000 vaccinees. Following the second dose, the SIR was 1.31 (95% CI, 1.05–1.62) with an estimated attributable risk of 0.86 cases per 100,000 vaccines [55]. The vaccine-related incidence of uveitis in Singapore was found to be six cases of uveitis among 431 patients (1.39%) after various vaccine (Pfizer-BioNTech and Sinopharm vaccines) [56]. A narrative literature review of 34 selected studies and five national databases investigated the incidence of uveitis after COVID-19 vaccination [57]. European Union data of the prevalence of uveitis was 0.3 among Pfizer recipients, 0.8 among Moderna recipients, 0.8 among AstraZeneca and 0.2 among Janssen. U.S. data demonstrated a prevalence of 0.2 among Pfizer/BioNTech recipients, 0.3 among Moderna, and 0.2 among Janssen. European Union data of prevalence (cases per million doses) of post-vaccination chorioretinopathy showed a prevalence of 0.03 cases per million among those receiving the Pfizer-BioNTech vaccine, 0.1 among those receiving Moderna, 0.1 from those receiving AstraZeneca, and none from Janssen. U.S. data of chorioretinopathy prevalence post-vaccination showed a prevalence of 0.01 among those receiving Pfizer-BioNTech, 0.01 from those receiving Moderna and 0.07 from those receiving Janssen [40]. Currently. available data show that nearly all available COVID vaccines can present with posterior uveitis including inactivated vaccines [58]. However, overall prevalence is too low and too variable to draw consistent correlation among specific vaccines.
    Bacille Calmette-Guerin (BCG) vaccine is used for prophylaxis against *Mycobacterium tuberculosis*. Rarely, patients presenting with systemic symptoms resembling bilateral panuveitis, chorioretinitis and/or optic neuritis have been reported [13, 59, 60]. Several cases of uveitis (panuveitis, multifocal choroiditis and VKH disease with serous retinal detachment) following tuberculin skin testing (purified protein derivative or PPD) have been documented [12, 61].
    Both anterior uveitis and panuveitis can occur in association with measles, mumps, and rubella (MMR) vaccination [11, 62]. Influenza vaccination has been associated with bilateral panuveitis, recurrent panuveitis, APMPPE and reactivation of ARN [10, 63–65]. Uveitis has also previously been documented following other vaccines, like hepatitis B, human papillomavirus, yellow fever, hepatitis A virus (HAV), and typhoid, and varicella vaccines [8, 66, 67].
    The possible causal mechanisms for the development of these post-vaccination uveitis are delayed-type hypersensitivity reaction and immune complex deposition following vaccination with the role of adjuvants in the immunologic process. Type III hypersensitivity reaction involving antigen–antibody complexes present in the aqueous humor may apply to the mechanism of COVID-vaccine-related uveitis [68]. There is also an implication of a vaccine-induced type I interferon secretion. The authors proposed that the vaccine mRNA activates RNA-sensing molecules including TLR3, TLR7, MDA5, and RIG-I which drive autoimmune processes in these patients.
    The possible cause of varicella zoster virus (VZV) reactivation following COVID-19 vaccination is induction of a strong T-cell response with increased CD8 + T cell and T helper type 1 CD4 + T cells. The VZV-specific CD8 + cells are temporally incapable of controlling VZV after the massive shift of naïve CD8 + cells. The other hypothesis of VZV reactivation is that aberrations in toll-like receptors (TLR) expression after vaccination induce of type I interferon (IFN-I) and potentiation of pro-inflammatory cytokines, which, may negatively modulate antigen expression [42, 69].
    - Intravitreal injection of antiangiogenic drugs; diethylcarbamazine.
    Brolucizumab (Beovu) is a novel single-chain antibody fragment that inhibits all isoforms of VEGF-A. It is the smallest of the anti-VEGF antibodies, with a molecular weight of 26 kDa, compared with 115 kDa for aflibercept and 48 kDa for ranibizumab, which have proven useful in the management of diabetic macular edema (DME). The KITE, KESTREL and KINGFISHER, phase III studies found intraocular inflammation in 2.2% to 4.7% of cases with brolucizumab (3-6 mg) versus 0.5% to 1.7% with aflibercept (2 mg). These studies report posterior involvement in the form of a retinal vasculitis in up to 1.6% [70–72]. Phase III trials of brolucizumab for neovascular age macular degeneration (AMD) also reported higher frequencies of intraocular inflammation (4.6%), including retinal vasculitis (2.1%) and RVO, compared to the aflibercept [73]. Although the cause of ocular inflammation with brolucizumab is unknown, the delayed onset (30–53 days) seems to signal an immune (Type III/IV hypersensitivity), rather than a toxic or infectious cause [73]. Rates of acute onset sterile inflammation have been reported to range from 0.05–2.1%, 0.05–1.1%, and 0.005–1.9% in aflibercept, bevacizumab, and ranibizumab, respectively with clinical manifestations of anterior chamber and/or vitreous cavity inflammation [74].
    Oral diethylcarbamazine (DEC) is a powerful microfilaricide used to treat onchocerciasis and it thought to be a possible cause of anterior uveitis, transient retinal pigment epithelial lesions, chorioretinitis, and optic nerve inflammation, which have been described previously in the literature [75–77].
  b Neoplastic conditions: leukemia and lymphoma associated posterior uveitis
    The prevalence of neoplastic inflammatory masquerade syndromes among the total population presenting with inflammatory intraocular disease was 1.8% in Rothova’s epidemiology study in the Netherlands and increased to 4.5% in patients older than 60 years [78].
    In cases of leukemia and lymphoma, ocular symptoms can be the presenting symptoms of systemic disease or its relapse after remission [7]. Some studies cite a prevalence of roughly 32 to 35% with the most common presentation being leukemic retinopathy [79–81]. It is important to note that leukemia and lymphoma-associated uveitis are part of the Uveitis Masquerade Syndromes (UMS) whereby the pathological process arises as a consequence of intraocular infiltration with malignant cells and is not secondary to an immune-mediated or infectious process. Among studies examining the prevalence of ocular manifestations of leukemia and lymphoma, an incidence of 20% has been reported [80].
    Leukemias (acute lymphoblastic leukemia (ALL), acute myeloid leukemia (AML)) are more commonly associated with anterior uveitis presenting with hypopyon [5, 6]. When the posterior segment is involved, it can be the result of direct invasion or secondary indirect effects of systemic disease. Malignant cells have been documented to infiltrate the uvea, optic nerve, cranial nerves, and peri-orbital tissues. Retinal hemorrhage, vitreous hemorrhage, vascular occlusion, and secondary infections all represent indirect sequelae of systemic malignant disease [7, 79–82].
    The designation of intraocular lymphoma includes primary intraocular lymphoma, mainly arising from the central nervous system (CNS) and secondary IOL, arising from outside the CNS as metastasis from a non-ocular neoplasm. Roughly 60–85% of primary IOLs will progress to involve the CNS [83–88]. Most lymphomas are low-grade B-cell lymphomas, with extranodal marginal zone B-cell lymphoma of MALT type (mucosa-associated lymphoid tissue) being the most common type. Follicular lymphoma also encompasses a high percentage of intraocular lymphoma [89]. Cases of marginal zone lymphoma and follicular lymphoma primarily involve the ocular adnexa [89, 90]. The other types of lymphomas include lymphoblastic lymphomas (of T-lineage and of precursor B-cell type), B-cell lymphoma, peripheral T-cell lymphoma, NK/T-cell lymphoma, classic Hodgkin lymphoma, Burkitt lymphoma, T-cell lymphoma, and NK-cell lymphoma, and these usually present with ocular adnexal involvement as well [90].
    Systemic Hodgkin lymphomas have been reported to be associated with secondary ophthalmic involvement in the form of bilateral panuveitis, anterior uveitis, vitritis, white chorioretinal lesions, papillitis and vasculitis [3]. Mucosa associated lymphoid tissue (MALT) lymphomas have been associated with retinochoroidal infiltration or panuveitis and secondary extramedullary location of acute myeloid leukemia can present as an anterior uveitis with anterior segment cell, dust-like pigmented keratic precipitate, iris bombe, ischemic bilateral retinal vasculitis and goniosynechiae, with associated retinal detachment and a sub-retinal space occupying lesion [2]. The issue with lymphomas is that usually a masquerade syndrome is suspected when intraocular inflammation is not responsive to steroids, although vitreoretinal lymphoma might initially be responsive to steroids [91].
    Other uveitis masquerade syndromes related to neoplastic causes include: uveal melanoma, retinoblastoma, bilateral diffuse uveal melanocytic proliferation, carcinomas metastatic to the eye, cancer-associated retinopathy (CAR), and melanoma-associated retinopathy (MAR) [92].
    For instance, isolated case reports have described peripheral uveal melanoma with clinical signs consistent with an anterior uveitis or panuveitis [93, 94]. Importantly, retinoblastomas can be misdiagnosed as pars planitis [95, 96] and Shields and al. reported that 3.9% of retinoblastomas were referred to their clinics as uveitis [97]. All-Ericsson et al. stated that the retinoblastoma’s growth pattern frequently results in clinical misdiagnosis, as there is often no apparent retinal mass and the presence of vitreous and anterior chamber seeding may mimic ocular inflammation [98]. Patients with metastatic esophageal cancer, breast cancer or adenocarcinoma of the lung can present with the initial diagnosis of idiopathic uveitis [99–101]. Interestingly, in Zhao et al.’ study, among patients whose diagnostic vitrectomy for etiology of uveitis initially unknown, 23% were lymphoma and 4% were metastatic carcinoma [102].
    Additionally, in Rothova et al. series of 1906 patients initially diagnosed with intraocular inflammatory disease, 116 (6%) patients were found to have a non-inflammatory cause of their ocular disorder, 36 (1.9%) with a neoplastic cause, and 52 (2.7%) with a non-neoplastic inflammatory masquerade syndrome. Only two (0.1%) had a paraneoplastic syndrome (one patient had cancer-associated retinopathy and the second patient had bilateral diffuse uveal melanocytic proliferation) [78]. Paraneoplastic vitelliform retinopathy can also mimic white dot syndromes [103].
  c Retinal vascular diseases: giant cell arteritis (GCA), ischemic ocular syndrome (IOS), and central retinal vein occlusion (CRVO)
    Giant cell arteritis (GCA) is a systemic granulomatous inflammatory vasculitis of medium and large vessels that leads to vision loss most commonly from arteritic anterior ischemic optic neuropathy (AAION) secondary to involvement of the short posterior ciliary arteries [17, 104]. This vascular occlusion results in ischemia to laminar and prelaminar segments of the optic nerve. Delayed choroidal perfusion on fluorescein angiography (FA) has been reported as a highly suggestive indicator of active GCA [105]. Case reports have demonstrated the presence of cotton wool spots (CWS) as an early manifestation of GCA (Fig. 3) [17], corresponding to localized accumulations of axoplasmic debris due to focal inner retinal ischemia [16, 106]. They have been reported as present in GCA even without other classic ocular findings, such as optic disc edema [107]. A recent study using Optical Coherence Tomography-angiography (OCTA) has shown focal areas of superficial and deep retinal capillary non-perfusion in eyes with AAION in the context of GCA, confirming delayed retinal perfusion, particularly in the peripapillary region [105]. A positive temporal artery biopsy (TAB) is the gold standard test for a histological diagnosis of GCA (Fig. 3a-d). Rai et al., have explained the etiology of CWS in CGA as likely being multifactorial: microembolization of platelets and/or hypoperfusion of terminal portions of retinal vasculature involved by giant cell arteritis [17].

**Fig. 3:**
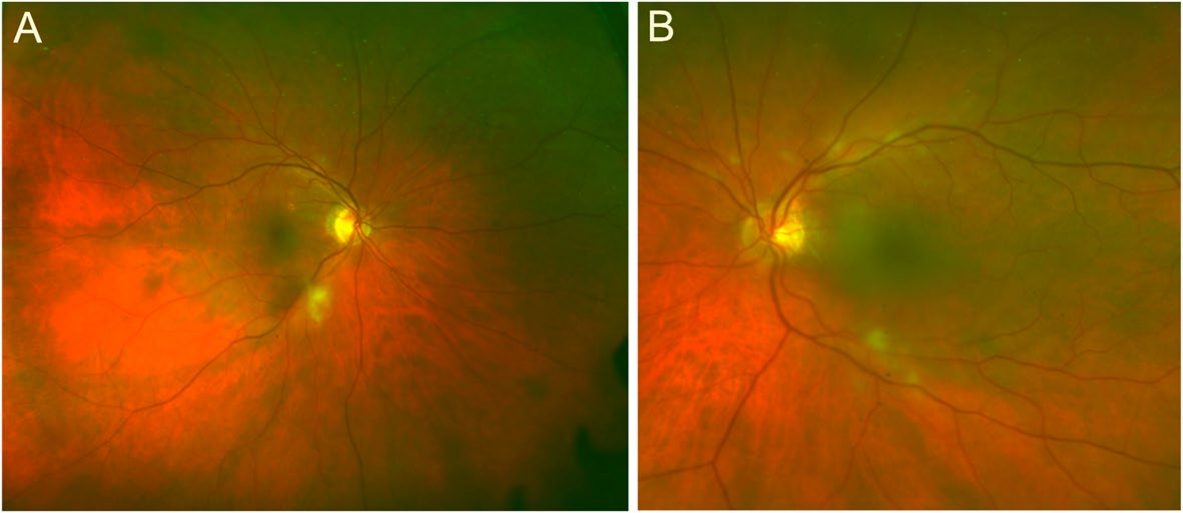
Fundus color pictures of a 75-year-old female with Giant cell arteritis (GCA) proven by a bilateral temporal artery biopsy. Note bilateral cotton-wool spots without optic disc edema seen in the right (**A**) and left (**B**) eyes
    The differential for CWS often includes ischemia (diabetes, hypertension, and retinal vein occlusion), neoplasia (leukemia and lymphoma), infections (human immunodeficiency virus), medications (interferon), and radiation retinopathy. However, the presence of CWS should prompt the clinician to include GCA in the differential diagnosis, especially when associated with the characteristic systemic symptoms of GCA, since this should prompt an urgent evaluation and initiation of systemic corticosteroids to prevent irreversible vision loss. Therapy should not be held pending TAB.
    Other retinal vascular diseases like ocular ischemic syndrome (IOS), central RVO and hypertensive retinopathy are well known by uveitis specialist to be among the differentials of uveitis.
    Vascular abnormalities also seen in ocular inflammatory diseases may be seen in the retina. These include capillary nonperfusion and ischemia, vascular occlusions, preretinal neovascularization, chorioretinal neovascularizations, microaneurysms and macroaneurysms, and telangiectasia [108]. Therefore, presence of subretinal and peripapillary neovascularization, macular telangiectasia type 2, macroaneurysm in combination with persistent myelin fibers have also been reported among the cause of nonneoplastic inflammatory masquerade syndromes [78].
  d Idiopathic eye-limited disorder not conforming to a defined syndrome:
    1. Central Serous Chorioretinopathy (CSCR) masquerading inflammatory exudative retinal detachment.
    Central serous chorioretinopathy (CSCR) is a common non-inflammatory condition affecting young adults and is characterized by neurosensory retinal detachment with or without pigment epithelial detachment (PED). CSCR is often listed as among the causes of nonneoplastic inflammatory masquerade syndromes in cohort studies [78]. CSCR is often caused by an abnormally high level of cortisol, induced by either exogenous use of steroids or endogenous hypercortisolism. A misdiagnosis of CSCR as uveitis leads to prolonged corticosteroid therapy with worsening of the condition. The lack of objective signs of inflammation such as retinal vasculitis, or optic disc hyperfluorescence on FA, or choroiditis on indocyanine green angiography (ICG) should point to a diagnosis of CSCR, as evidenced by Papadia et al. [109–111].
    Diffuse pigment epitheliopathy, a severe form of CSCR, presents with multiple large RPE detachments mixed with possibly subretinal serofibrinous exudates. FA shows early hyperfluorescent foci of dye leakage from the choroid, with late staining of the surrounding retina. These leaking points often represent large hyperfluorescent patches with multiple hyperpermeable areas in the choroid that can be misdiagnosed for: VKH, diffuse uveal effusion syndrome, choroiditis, or lymphoma [112].
    EDI-OCT shows serous retinal detachments with hyperreflective subretinal material in diffuse pigment epitheliopathy. However, unlike a retinal detachment in inflammatory choroiditis, there is no subretinal septae or multilobular pooling [113–115]. The differential includes multifocal choroiditis, such as presumed ocular histoplasmosis syndrome, which may be associated with an exudative macular detachment in association with choroidal neovascularization. EDI-OCT also classically demonstrates a thickened choroid which is a hallmark off this condition. Posterior cystoid retinal degeneration in chronic CSR may also mimic a cystoid macular edema from various causes.
    d.2. Rhegmatogenous retinal detachment (RD) masquerading as exudative panuveitis with anterior chamber inflammatory reaction.
    Typically, an exudative retinal detachment (RD) is associated with various forms of ocular inflammatory diagnoses: VKH syndrome, posterior scleritis, sympathetic ophthalmia, panuveitis, multifocal choroiditis (MFC) with panuveitis, posterior uveitis and necrotizing scleritis [116]. However, rhegmatogenous RDs can rarely present with features of inflammation such as Schwartz-Matsuo syndrome. Canonically, Schwartz-Matsuo is associated with increased intraocular pressure along with anterior chamber inflammation, often believed to be due to liberated photoreceptors from retinal breaks migrating to the anterior chamber, mimicking anterior chamber cells and occluding the trabecular meshwork [117, 118]. Apart from the Schwartz-Matsuo syndrome, rhegmatogenous RDs can also present with anterior chamber inflammation and exhibit features usually seen in uveitic serous RDs, specifically diffuse choroidal thickening, choroidal detachment, and/or white blood cells as well as fibrin in anterior chamber [119].
    There are a couple of reports of chronic rhegmatogenous RD presenting as a panuveitis with anterior uveitis and hypotony [119, 120]. The visualization of retinal breaks is sometimes difficult. For instance, preoperative undetected retinal tears are not rare findings in routine rhegmatogenous RD management when the retinal breaks are positioned anteriorly. Despite clear visualization of the fundus, it has been reported that retinal breaks are not found in 2.2% to 4% of phakic rhegmatogenous RDs [121]. Identifying underlying breaks in the setting of severe inflammation can be even more challenging due to posterior synechiae and hazy media [119]. The lack of visualization of a retinal break should not preclude its possible existence when examining the undifferentiated patient with purported intraocular inflammation.
    d.3. Coats disease
    Coats disease is a rare idiopathic telangiectatic neovascular disease of the retina. The definite diagnosis of Coats disease can be challenging especially in its advanced stage, as it could mimic other ophthalmic conditions, such as retinoblastoma but also posterior uveitis. Coats disease is among the differential diagnoses for leukocoria, intermediate and posterior uveitis, and multifocal non-infectious exudative RDs, especially in young patients [122]. When diagnosed in the adult population, its manifestations are often smoldering presenting as persistent floaters and visual distortion. Shields et al., in the 2000 Sanford Gifford Memorial Lecture, described the clinical features [123]. The authors described a clear definition to diagnose true idiopathic Coats disease and differentiate from other simulating conditions. They defined Coats disease as idiopathic retinal telangiectasia with intraretinal and/or subretinal exudation without appreciable retinal or vitreal traction. Moreover, the diagnosis of Coats disease should not be accepted in an adult until other causes of exudative retinopathy are clearly excluded. Coats disease is a sporadic nonhereditary condition that is not associated with identifiable systemic abnormalities. There is no predilection of Coats disease for race with 76% of patients being males. The anterior segment findings are usually normal. The majority of the telangiectasias are located in the inferior and temporal quadrants between the equator and the ora serrata, with about one third extending posterior to the equator toward the vascular arcades. Telangiectasia in the macular area is uncommon. The telangiectasia is usually confined to the aforementioned quadrants, but the exudation is more widespread. Retinal bleeding and vitreous hemorrhage are unusual in Coats disease [124]. Optic disc and retinal neovascularization are also uncommon. See Supplemental Table for Coats disease’s diagnosis. Thus, these varied presentations make its diagnosis challenging. However, there are specific features of Coats disease that do allow for its differentiation from other uveitic entitites. More specifically, the unilateral nature of retinal vessel telangiectasias and lightbulb aneurysms typically in the absence of vitritis, the presence of yellow exudates and fundus fluorescein angiography that capture the characteristics areas of leakage from telangiectatic vessels and capillary nonperfusion can all point towards a diagnosis of Coats disease [125].
    d.3. Hereditary retinal diseases and vitreoretinopathies
    Hereditary retinal diseases (retinitis pigmentosa, macular dystrophy, TRAPS (TNF-receptor-associated periodic syndrome)) represented 31% of the causes of nonneoplastic inflammatory masquerade syndromes in the cohort study by Rothova et al. and they exhibit the.longest time to diagnosis, specifically 42 weeks (range 0–156 weeks) [78]. Among other conditions, autosomal dominant neovascular inflammatory vitreoretinopathy (ADNIV) is a rare autoimmune condition that presents as uveitis and vitreoretinal degeneration. The condition progresses to retinal degeneration, peripheral arterial closure, peripheral retinal neovascularization, tractional retinal detachment and neovascular glaucoma. ADNIV is caused by mutation in *CAPN5* [126, 127]. Furthermore, familial exudative vitreoretinopathy (FEVR) of childhood is sometimes misdiagnosed as uveitis (Fig. 4). FEVR is an inherited disorder characterized by retinal traction, peripheral vitreous opacities, and subretinal and intraretinal exudates [128]. Conversely, retinitis pigmentosa-like retinal pigmentary changes are attributed to chronic uveitis [129]. Uveitis is a rare feature of VCAN-related vitreoretinopathy which includes Wagner syndrome and erosive vitreoretinopathy (ERVR), and which is characterized by "optically empty vitreous”, myopia, cataract, night blindness associated with progressive chorioretinal atrophy, retinal traction and retinal detachment in the advanced stages of disease [130]. The posterior segment presents with changes of the retinal pigment epithelium and overlying retina (pigment condensation, vascular sheathing, pigmented lattice degeneration, and later chorioretinal atrophy in the retinal periphery). VCAN-related vitreoretinopathy is autosomal dominant [131].

**Fig. 4:**
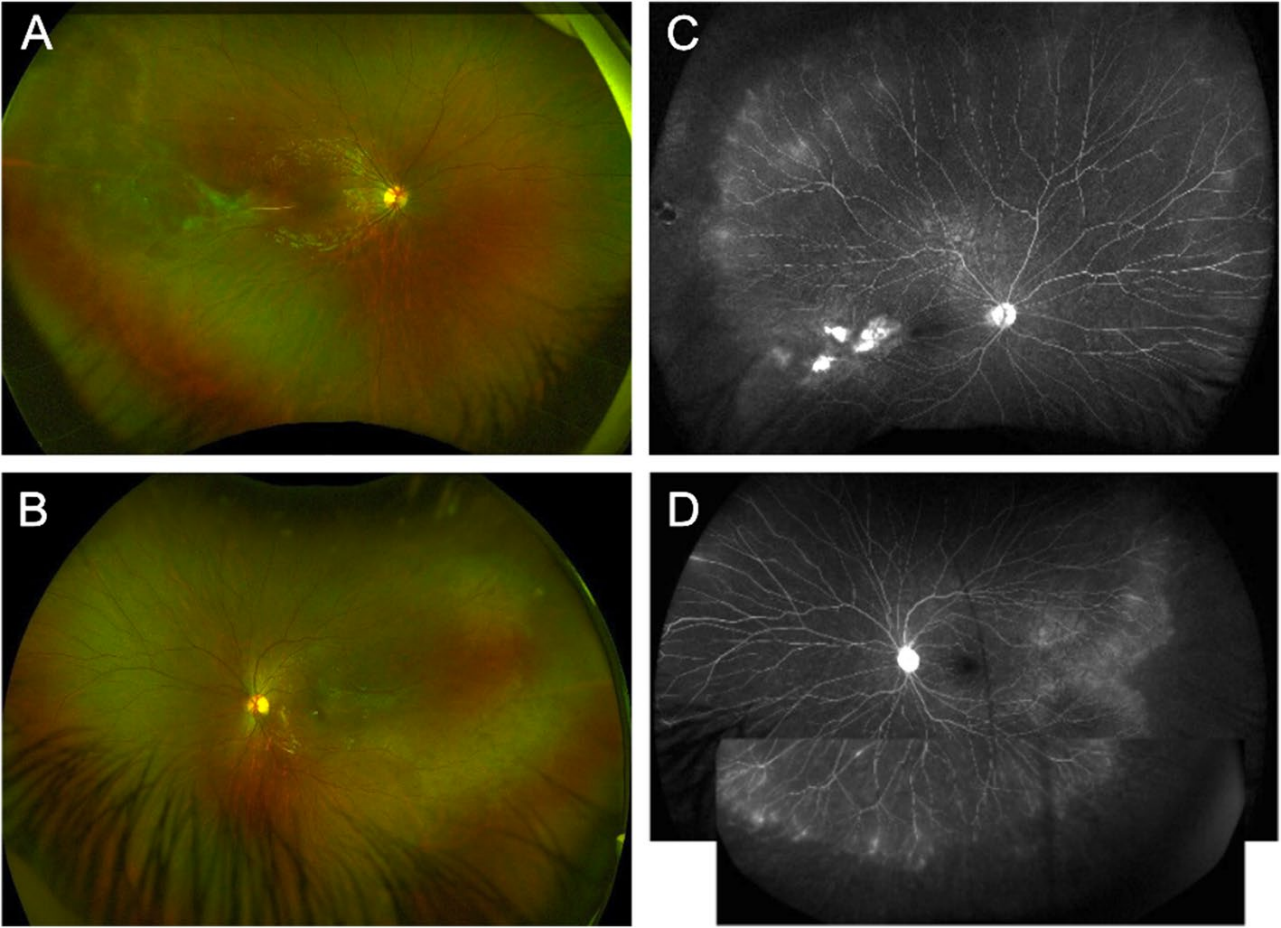
Multimodal image of a patient with familial exudative vitreoretinopathy. Wide field Optos fundus photos of the right (**A**) and left (**B**) eye show perivascular sheathing, worse on the right eye. Fluorescein angiogram shows focal venous leakage with staining of lesions temporal to the macula (**C**). Both the right and the left eye show diffuse peripheral leakage with capillary dropout in the periphery (**D**)
    Moreover, subretinal scarring in autosomal recessive bestrophinopathy (ARB) can mimic chorioretinitis. ARB is a specific and recognizable phenotype that can be differentiated clinically from other overlapping clinical syndromes or retinal dystrophies, without prior knowledge of the inheritance pattern, or genotype [132].
    d.4. Other: Myopic degenerations, drusen
    A large cohort study of 111 patients previously diagnosed with MEWDS were reviewed and showed that 26% of patients were subsequently given an alternative diagnosis, including other posterior uveitis, primary vitreoretinal lymphoma, myopic degeneration, and central serous chorioretinopathy [133].
    In punctate inner choroidopathy (PIC), stage II lesions appear as a focal elevation of the RPE with corresponding disruption of the inner and outer segments of the photoreceptor interface using spectral-domain (SD)-OCT. Therefore, PIC lesion can mimic drusen that are found in Bruch's membrane and may represent precursors for the development of age-related macular degeneration [134].
  e Systemic disorders
    1. Purtscher-like retinopathy as a presentation of Systemic lupus erythematosus (SLE)
    Purtscher retinopathy is characterized by patches of retinal whitening and hemorrhage around the optic nerve and in the posterior pole which are classically identified in patients who have suffered from severe trauma, including long-bone fracture, cephalic or thoracic compression, and crush injury (Fig. 5). Purtscher-like retinopathy may occur due to acute pancreatitis, renal failure, collagen vascular diseases, hemolysis, elevated liver enzymes, low platelets (HELLP syndrome), and multiple myeloma [135]. Purtscher-like retinopathy is also a rare and severe complication of systemic lupus erythematosus (SLE) or as a distinct category of severe retinal vasocclusive disease in SLE [136]. The pathogenesis of Purtscher-like retinopathy associated with SLE is not fully understood. It is likely due to the formation of microemboli that results in retinal vascular occlusion and microvascular infarcts of the retinal nerve fiber layer [135]. Some authors have suggested that precapillary arteriole occlusion is followed by altered retinal microvascular permeability [137]. SLE-related eye involvement can be diagnosed in approximately one-third of the patients and is usually indicative of disease activity [138, 139]. SLE retinopathy, either unilateral or more often bilateral, is responsible for visual loss secondary to vasculitis of the retinal capillaries and arterioles (Fig. 4). Interestingly, a reduction in retinal vessel density measured by Optical Coherence Tomography (OCTA) but also seen on FA may be a good marker of SLE activity.
    e.2. Amyloidosis

**Fig. 5:**
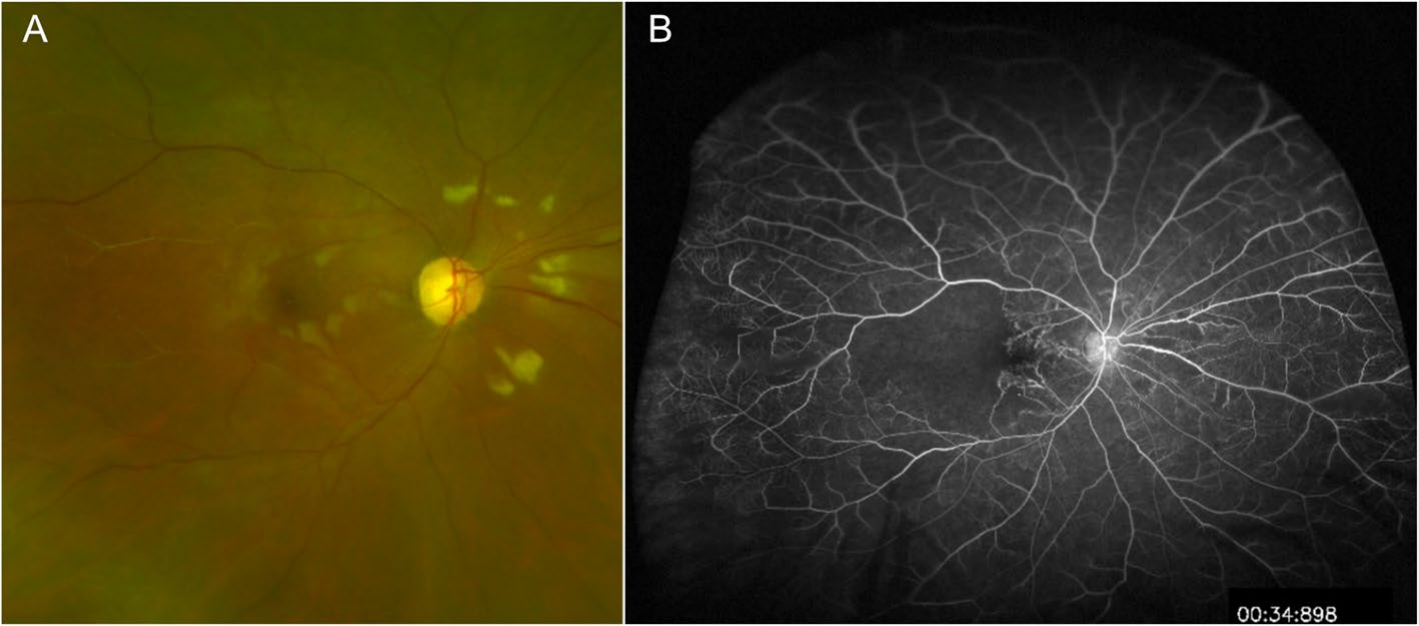
Fundus color picture of a 52-year-old female with systemic lupus erythematosus and lupus nephrosis. She was diagnosed as a case of lupus retinopathy owing (**A**) to patches of polygonal retinal whitening nasal to the nerve and on the fovea and (**B**) occlusion vasculitis of the retinal capillaries and arterioles on fluorescein angiography
    Familial transthyretin amyloidosis (FTA) is a rare and severe autosomal dominant disease that is caused by a mutation in the transthyretin (TTR) gene.
    Ocular manifestations of amyloidosis are found in 10% of patients, presenting as deposition of amyloid in the lacrimal glands, conjunctiva (abnormal conjunctival vessels), lens capsule, iris epithelium, ciliary pigment epithelium, cornea (loss of corneal sensitivity and neurotrophic corneal ulcers; keratoconjunctivitis sicca), chronic open-angle glaucoma, optic neuropathy and vitreous (vitreous deposits adhering to the posterior lens capsule, as pseudopodia lentis) [140, 141]. The appearance of amyloid in the vitreous has been described as sheet-like, film-like, band-like, cobweb-like, glass wool-like, cotton-like and stringy fibril-like [142]. Retinal amyloid angiopathy presents with microaneurysms, retinal hemorrhages, pinpoint white deposits, needle-shaped deposits, retinal cotton-wool spots and retinal ischemia of variable extent with amyloid deposition in the vitreous fluid (Fig. 6) [143–145]. Retinal changes occur in about 20% of patients and is more prevalent in patients with Y114C mutation [146]. Choroidal amyloid angiopathy has also been described in the form of late hyperfluorescence on ICG along the choroidal vessels (Fig. 6E) [147, 148]. Transthyretin amyloidosis may be misdiagnosed as any posterior uveitis with vitreous opacities resulting in a significant diagnostic delay [149, 150].

**Fig. 6:**
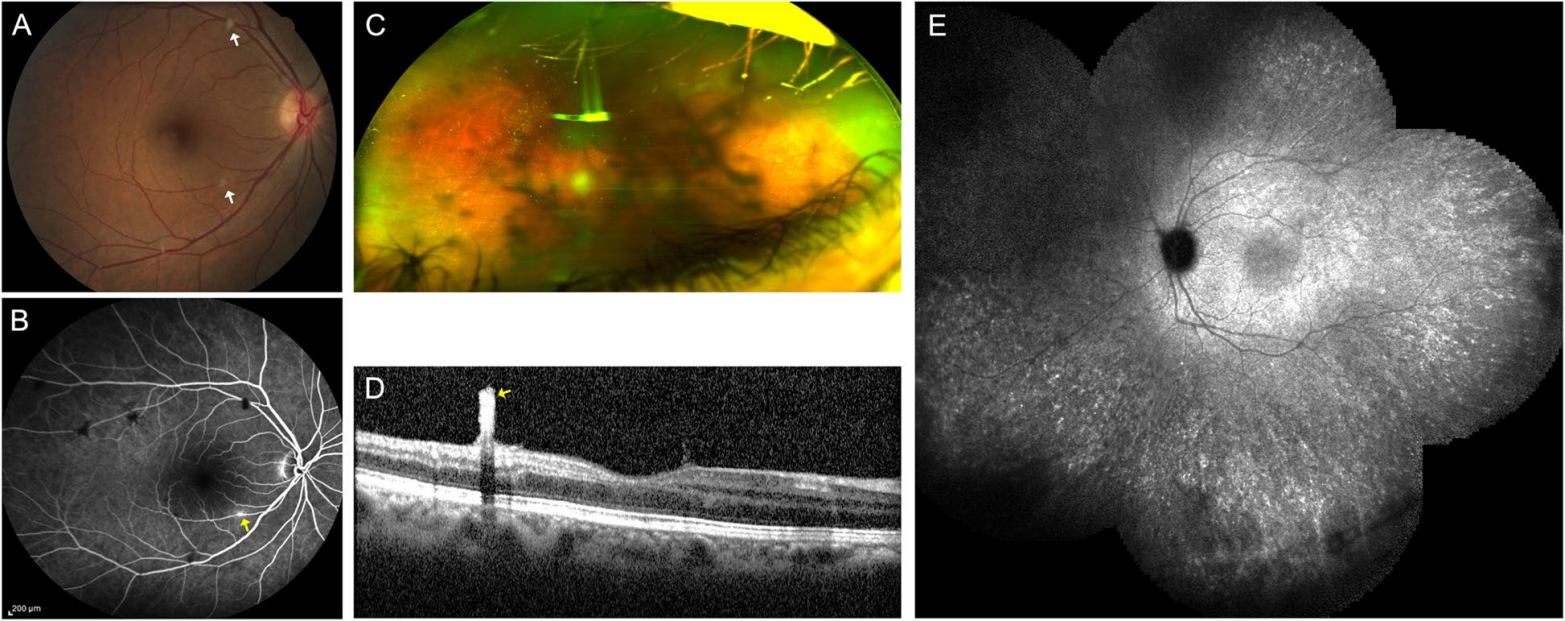
Fundus color picture of a 46-year-old female with transthyretin amyloidosis. She initially was referred for blurry vision as a case of intermediate uveitis (vitritis) and retinal vasculitis in the right eye and a pars plana vitrectomy was performed in the right eye. She later presented with a left eye involvement. The pictures demonstrate retinal vascular sheathing as shown in arrows in the right eye (**A**) and intermediate uveitis with vitritis in the left eye (**B**). The genetic diagnosis of transthyretin amyloidosis was made. Fluorescein angiography showed focal vascular staining in intermediate phase (arrow) (**C**). The Spectral Domain OCT (SD-OCT) showed a deposit perpendicular to the retinal surface towards the vitreous from the retinal vessel (arrow) (**D**), and hyperfluorescent foci and staining along choroidal and retinal vessels in the supero-, infero- and temporal periphery on ICG in late phase (**E**)
2. Misdiagnoses among Uveitis entities
  a Infectious
    - Acute Retinal Necrosis (ARN) misdiagnoses
    There are two main viruses that cause this condition: the varicella zoster virus (VZV) and the herpes simplex virus (HSV). Other infections can mimic ARN, mainly: cytomegalovirus retinitis, syphilis, toxoplasmosis, infectious endophthalmitis, ocular aspergillosis, and primary ocular lymphoma.
    - Syphilis (sectoral retinitis)
    Syphilis is a particularly important differential diagnosis to keep in mind when considering the possible etiology in a case of retinitis. Recent studies suggest a trend of increasing syphilis incidence. Per the Centers for Disease Control in the United States (CDC), the current incidence of all stages of syphilis in 2018 amounts to 35.3 cases per 100,000 and 10.8 cases of either primary or secondary stages per 100,000. This is from a nadir of 11.2 and 2.1 respectively in 2000 [151].
    Acute syphilitic posterior placoid chorioretinitis (ASPPC) that presents as a large, yellowish, circular or oval, placoid lesion at the level of the RPE in or near the macula [152]. But one must keep in mind that syphilis has variable manifestations and can mimic many other inflammatory diseases, both infectious and autoimmune. Its nickname as ‘the great impostor” is well-earned [153]. A retrospective observational case series of patients who had intraocular inflammation due to syphilis over a 15-year period showed that presentations included: isolated anterior non-granulomatous uveitis, intermediate uveitis, panuveitis, papillitis, placoid chorioretinitis and frank retinitis. Among those with a late diagnosis, spreading retinitis was observed. Syphilitic retinitis can mimic a viral ARN. Although it is easy to identify syphilis-related placoid lesions as syphilis-related, retinitis lesions can be difficult to relate to syphilis as the differential for retinitis is broad, including large retinochoroiditis secondary to toxoplasmosis, viral ARN, and fungus endophthalmitis among others. Specific features of syphilitic retinitis have been described to narrow the differential of retinitis. These include superficial retinal precipitates*,* inner retinitis*,* and outer retinitis [154, 155]. Patients may also display yellow-white patches of diffuse retinitis and coalescent posterior pole retinal whitening representing necrosis [156]. Retinal vascular sheathing may also be noted, representative of occlusive pathology with retinal ischemia due to endarteritic changes in the setting of syphilis [157]. Another pattern of disease occasionally reported is a punctate retinitis limited to more peripheral retina that is otherwise interpreted as miliary lesions. They are small round to oval, yellow retinal lesions, measuring less than one-fourth of a disc diameter size, with distinct margins, involving complete thickness of retina on OCT, in a pillar like manner, associated with ground glass retinitis, and an outer retinal placoid lesion or with retinal vasculitis [158, 159]. These lesions are of particular interest because they may be characteristic of ocular syphilis but misdiagnosed as another uveitis entity [158].
    - Retinochoroiditis secondary to Toxoplasma gondii
    Toxoplasmosis is the most common cause of posterior uveitis in many countries and presents commonly as a typical retinochoroiditis with unilateral focal retinitis at the border of a preexisting pigmented retinochoroidal lesion and overlying vitritis [160]. In immunocompetent patients older than fifty and in immunocompromised patients, the toxoplasmic infection remains an important cause of posterior/panuveitis and its presentation in those populations are notably atypical. These atypical lesions consist of large areas of retinal necrosis or retinochoroiditis without adjacent preexisting pigmented retinal scar or retinochoroiditis in both eyes [160–164]. The value of performing an anterior chamber paracentesis for laboratory evidence of ocular toxoplasmosis in the aqueous humor is paramount in differentiating *T. gondii* retinochoroiditis from similar lesions in immunocompromised or immunocompetent older individuals. The diagnosis of ocular toxoplasmosis can be made by calculating the Goldmann-Witmer coefficient (GWC), or by polymerase chain reaction. When atypical, it might be of interest to ask for a serological evidence of exposure to the parasite [160–164]. Serum is tested for estimation of IgM and IgG antibodies with *T. gondii* IgG antibody appearing in the serum 2–3 weeks after acute infection.
    - Presumed Tuberculous Serpiginous-Like Choroiditis (Tb-SLC) and Multifocal Serpiginoid Choroiditis masquerading serpiginous choroiditis (SC)
    SC is a rare, recurrent, idiopathic ocular disease leading to severe inflammatory damage of retinal pigment epithelium (RPE), choriocapillaris, and choroid.
    It is essential to differentiate the tuberculous presentations of ocular tuberculosis, i.e. presumed tuberculous serpiginous-like choroiditis (Tb-SLC) and multifocal serpiginoid choroiditis (MSC) from classic SC because treatment with an immunosuppressive therapy can have devastating consequences in the case of concomitant tuberculous infection.
    Patients with Tb-SLC come from highly endemic regions, and some clinical features in favor of presumed Tb-SLC rather than SC, include significant vitritis, multifocal lesions, and serpiginous lesions in the posterior pole and periphery [165]. Cases of SC, in contrast, reveal minimal or no vitritis and frequently show bilateral involvement with larger solitary lesions extending primarily from the juxtapapillary area and sparing the periphery [166]. Bansal et al., reported that in eyes with Tb-SLC, vitreous inflammation was present in 81% of eyes, multifocal lesions in 94%, and noncontiguous to optic disc in about 87% of eyes [165].
    Interestingly, it has been shown that OCT may help differentiate between Tb-SLC and SC since vitreous hyper-reflective spots, intraretinal fluid, sub-RPE drusenoid deposits, and choroidal granulomas on OCT images may indicate Tb-SLC [167].
    - COVID
    Classically, COVID-19 infection spreads via respiratory droplets and primarily impacts the respiratory tract, resulting in severe acute respiratory syndrome. Data showing the presence of coronavirus in affected subjects’ tear samples implicates a correlation between COVID infection and ophthalmic symptoms [168–170]. Subsequent studies have shown a wide variety of ophthalmic manifestations; the most common being conjunctivitis [171, 172]. Retinochoroidal involvement is rare with isolated case reports demonstrating the presence of acute retinal vasculitis, neuroretinitis and panuveitis, toxoplasma retinochoroiditis, pars planitis, VKH, MEWDS, PIC, and retinopathy led by COVID-19 inflammatory syndrome or reactivation of previously quiescent uveitis, in the setting of acute COVID infection [42, 44, 173–181]. The hypothesis for posterior segment inflammation in the eye involves COVID-19 related inflammation of endothelial cells using the angiotensin-converting enzyme 2 receptor and immune-mediated inflammation which eventually result in ischemic retinal vasculitis [182]. Furthermore, viral RNA of COVID-19 has been detected in the retina of affected patients [183, 184].
  b Non infectious
    1. White dots syndromes misdiagnoses.
    There are specific diagnoses thought to be related to MEWDS: acute idiopathic blind spot enlargement syndrome (AIBSE), acute zonal occult outer retinopathy (AZOOR) which can affect the retina around and away from the disc; acute macular neuroretinopathy with unilateral para-central scotoma; and also cancer and melanoma-associated retinopathies (CAR). Below we will focus on various entities that can mimic the white dots syndromes:
    b.1.1. Acute Posterior Multifocal Pigment (APMPPE) misdiagnosed for Vogt-Koyanagi-Harada (VKH) disease.
    APMPPE is an immune-driven, rare inflammatory eye disease. Choriocapillaris hypoperfusion has been recently described as the primary event in the pathogenesis of APMPPE with the hypothesis being that, in APMPPE, an isolated disruption to the choriocapillaris leaves the choroid in a sufficiently functional state to largely maintain the RPE/photoreceptor integrity [185–187].
    Some APMPPE cases may have overlapping findings with VKH. In such atypical APMPPE cases, inflammation may manifest as a retinal detachment at the level of photoreceptor inner segment myoids that is named as a bacillary layer detachment [188]. This phenomenon has also been previously documented in VKH [189] and in macular toxoplasmosis chorioretinitis with the hypothesis that degenerating cone photoreceptors are capable of shedding their inner segments and that patients with preexisting pachychoroid spectrum disease may manifest a more significant retinal fluid accumulation in the setting of superimposed chorioretinal inflammation. In sum these phenomena result in a bacillary layer detachment. Li et al. and Ketamura et al. have described case reports with unilateral fundus placoid and typical FA findings for APMPPE [190, 191]. On OCT however, patients had cystic retinal detachments and outer retinal disruption that was more suggestive of VKH than APMPPE. However, the absence of the ‘starry sky’ appearance that is typically seen in VKH suggested instead an atypical presentation of APMPPE. In our experience we have also encountered similar atypical APMPPE findings with a detachment that apparently separated the photoreceptor inner segment myoids from inner segment ellipsoids.
    b.1.2. Other mimickers for Vogt-Koyanagi-Harada (VKH) disease
    A wide range of ocular conditions can mimic VKH, especially sympathetic ophthalmia which is its main competing diagnosis in patients with a history of ocular trauma or previous intraocular surgery. Other syndromes misdiagnosed as VKH are: uveal effusion syndrome; posterior scleritis; AMPPE; and MEWDS. Posterior scleritis can also present with ocular pain, hyperemia and increased choroidal thickness with serous retinal detachments on OCT. However, it is usually unilateral and not associated with neurologic signs or cutaneous findings. B-Scan ultrasonography remains an important modality which may show the classic but non-pathognomonic T-sign, which represents fluid collection in the sub-Tenon space and the optic nerve sheath, with variable degree of thickening of choroid and sclera [109].
    VKH itself is one of the most common causes of misdiagnosis of CSCR. The similarities between the two conditions are subretinal detachment, leakage from RPE in FA as well as bullous retinal detachment in certain atypical CSCR [109]. FA shows multifocal pinpoint leaks in both, but less numerous in CSCR and with the addition of late prominent pooling in the area of subretinal fluid and late optic disc staining in VKH [109]. ICG demonstrated multiple areas of choroidal hyperpermeability in CSCR, in contrast to VKH, where it demonstrates diffuse choroidal hyperpermeability and hypofluorescent dark dots [192, 193]. FAF shows diffusely speckled hyperautofluorescence related to longer duration of CSCR, a feature not seen in VKH [194].
    b.1.3. Choroidal granulomas (CGs) in sarcoidosis masquerading like birdshot chorioretinopathy lesions (BC) lesions on Indocyanine Green Angiography (ICG)
    Choroidal granulomas (CG) in the absence of anterior uveitis are rare but well-recognized manifestation of sarcoidosis, occurring in approximately 5% of patients with ocular sarcoidosis [195]. CGs manifesting as the sole lesion in ocular sarcoidosis has been previously described [195–198]. Desai et al. reported on the largest case series of either solitary of multifocal CGs in nine patients with biopsy-proven sarcoidosis [195]. The typical CG shows hyperfluorescence on FA with late staining. ICG is an important imaging modality for the identification of CGs in sarcoidosis because it identifies sarcoid CGs before other clinical signs of the disease [199]. Four different ICG patterns have been described. The first pattern is hypofluorescent irregularly distributed choroidal lesions in the mid-periphery. These lesions are seen in both early and intermediate phases of ICG and are not readily visible on fundus photography or FA [199]. The second pattern is focal hyperfluorescent pinpoints in intermediate and late phases of ICG. The third pattern is fuzzy choroidal vessels with leakage in the intermediate phase of ICG. This finding represents the vasculitic changes commonly seen in sarcoidosis. The fourth pattern seen is diffuse late zonal choroidal hyperfluorescence with late staining [200–202]. EDI-OCT also has a role in the diagnosis of choroidal granulomas. In a series of 44 cases of CGs of various causes, Invernizzi et al. have shown that the EDI mode could visualize 100% of CGs detected on ICG. All CGs showed increased transmission of the OCT signal as compared with the surrounding choroid [203]. EDI-OCT may also be more sensitive than ICG in detecting early variations in the size of choroidal granulomas in response to treatment [204]. Furthermore, the location of CG (optic disc nodules and/or solitary choroidal nodules) can be suggestive of a sarcoidosis rather than other hypofluorescent lesions on ICG like BC (≥ 3 peripapillary birdshot lesions are required for diagnosis) [205, 206].
    b.1.4. Presentations of birdshot Chorioretinopathy (BC) with minimal or absent birdshot spots.
    Birdshot chorioretinopathy (BC) is a uveitis predominantly affecting the posterior segment of the eye with dual, independent retinal and choroidal inflammation [207–210]. BC can be expected to be associated with HLA-A29 positivity almost 100% of the time. This rate is by far the highest known HLA association with a disease. Retinal vasculitis of small and large vessels, profuse leakage of fluorescein into the retina and hypofluorescent dark dots on ICG, are the classic multi-modal features observed early in the disease. However, BC can present without classic fundus findings, lending BC to masquerade as other pathologies. Recently, Herbort et al. have highlighted that in the early stage of disease, BC lesions are not disease defining [207], and relying on their presence leads to diagnostic delay when early diagnosis and treatment are important [211]. Indeed, hypofluorescent dark dots on ICG can be present without BC fundus lesions, indicating early disease before cicatricial depigmentation has occurred. This relies on the fact that hypofluorescent dark dots on ICG are not the angiographic expression of BC fundus lesions but rather correspond to previously active stromal choroiditis having left depigmented cicatricial areas where pigment has been destroyed by the inflammatory mechanism [207]. Occasionally, early-stage BC can be highly asymmetrical, and mistaken for intermediate uveitis, and can also be mimicked by ocular sarcoid.
  c Others
    1. Extensive scarring throughout the fundus: progressive subretinal fibrosis and uveitis syndrome (PSFU).
    Progressive subretinal fibrosis and uveitis syndrome (PFSU) is a rare idiopathic inflammatory disease that classically starts as multifocal choroiditis followed by large areas of subretinal fibrotic lesions. The disease is often misdiagnosed in early stages due to the many differential diagnoses of early lesions, which can include: APMPPE, POHS, MEWDS, PIC, MFC, Acute Retinal Pigment Epitheliitis (ARPE), retinal necrosis, sympathetic ophthalmia and variable amounts of anterior segment and vitreous inflammation among others. [212–216]. Clinically, PFSU can be distinguished from PIC lesions in that anterior chamber inflammation and vitritis are more significant and fundus lesions are larger [217, 218]. The inexorable progression of lesions in PSFU to dense fibrosis and later atrophy is also qualitatively different [219, 220]. While it is commonly bilateral and affects adults, reports of unilateral and pediatric cases have been published [220, 221]. Seemingly, the disease’s end-stage phenotype is the only common thread among published divergent cases.
    c.2. Sarcoid Choroidal Granulomas presenting as Paving Stone lesions
    Sarcoidosis typically affects the eyes in the form of both anterior, posterior and pan-uveitis. Peripheral sarcoid lesions can be mistaken for pavingstone degenerative lesions that are commonly seen on dilated fundus exam, though upon closer examination there are clear clinical differences. Multiple chorioretinal peripheral lesions or small clustered yellowish lesions located in the peripheral infero-temporal retina are suggestive of sarcoidosis. The consensus workshop of an international committee for diagnostic criteria for ocular sarcoidosis have shown that multiple chorioretinal peripheral lesions (active and/or atrophic) are suggestive of ocular sarcoidosis [222]. These white lesions can be the earliest manifestation of sarcoidosis, even when systemic work-up is negative for the disease, and they differ from late pavingstone lesions in that they are fine looking, smaller, and lack atrophic changes [223].
    c.3. Drug related. Paradoxical inflammatory effects of anti-Tumor necrosis factor α (TNFα) and uveitis
    Paradoxical inflammatory effects of anti-Tumor necrosis Factor-α (anti-TNFα) have been noted most probably because of a disequilibrium in cytokine balance and include exacerbation or initiation of drug-induced autoimmune diseases, and uveitis. Etanercept is the most commonly implicated drug and patients with spondylarthropathies are more commonly affected by this adverse reaction. However, there have been documented cases in patients with rheumatoid arthritis, psoriatic arthritis and juvenile idiopathic arthritis. There are also select cases published implicating adalimumab, and infliximab [36, 39, 224–228]. With etanercept, uveitis, scleritis and a sarcoidosis-like syndrome with ocular granulomas have been reported [225, 229]. Infliximab has been linked to uveitis, and sterile endophthalmitis. 
    Adalimumab has been found to paradoxically cause retinal toxicity [225].
    Etanercept is significantly more likely to be associated with uveitis than either infliximab (odds ratio 5.375) or adalimumab (odds ratio 8.6). Etanercept is known for worsening the uveitis course or even for inducing inflammation, as a paradoxical effect [230].
    Etanercept is a dimeric protein that is part-TNF-a receptor and part-Fc molecule of IgG. It prevents TNF from binding to cell-surface receptors. Etanercept is unique in the anti- TNF α medication category in that it preferentially inhibits the TNFα receptor, acting as a decoy. Unlike infliximab and adalimumab, which preferentially inhibit the free-floating soluble TNF α molecule. Several theories exist as to why etanercept causes intraocular inflammation, contrary to its TNF α counterparts, it does not inhibit interferon gamma (IFN-γ) which has been shown to cause intraocular inflammation, scleritis, and a sarcoidosis-like syndrome.
    Moreover, the relationship between anti-TNF α and induction of optic neuritis associated with demyelinating diseases remains unclear, but in several cases the etiology suggests both golimumab and certolizumab.

## Conclusions

In this review, we described both ophthalmic pathology that can falsely mimic posterior uveitis as well as often under-considered etiologies of posterior uveitis. We highlighted posterior uveitis mimickers that are not included in the group of immune-mediated uveitis entities, classically identified as *“Uveitis Masquerade Syndromes”*.

Our review yields several important takeaways. In cases of recurrent or persistent ocular inflammation, or in cases with an unclear history, it is important to rule out underlying neoplastic disease, as these entities often require different treatment, and may carry systemic implications along with implications on patient survival.

Additionally, it is always important to consider nonmalignant, mimickers of posterior uveitis. Inherited retinal conditions, such as retinitis pigmentosa and the vitreoretinopathy, FEVR, can mimic chronic posterior and intermediate uveitis. Conversely, a rare, heterogeneous, group of inherited vitreoretinopathies (ADNIV, VCNA-related vitreoretinopathy) and inherited retinal diseases can present with uveitis and vitreoretinal degeneration. This highlights the overlapping features of these conditions with more common etiologies of posterior uveitis and the importance of genetic testing. The ability to make a correct diagnosis is crucial for patient treatment as well as genetic counseling.

Having a high suspicion for infectious uveitis is also crucial, since the correct diagnosis and treatment can potentially prevent rapid vision loss, such as in ARN, serpiginous-like tuberculosis, *T. gondii*, and syphilis. Misdiagnosing one of these infectious conditions and starting an immunosuppressive treatment may lead to exacerbation of tuberculous infection and even death. The lack of accurate diagnosis in some uveitis entities like serpiginous-like tuberculosis makes it difficult to differentiate with idiopathic SC, especially in non-endemic areas for tuberculosis. The distinct location of the fundus lesions is the only clear way to decipher between the two entities. This is particularly confusing since the angiographic pattern is similar in both classic SC and presumed Tuberculosis-SLC and the labs/imaging are often negative except for positive interferon-gamma (IFN-γ) release assay or tuberculin skin test results in the latter entity.

In a systemic disease like in SLE, the clinical inflammatory features can be difficult to detect. A noninflammatory, Purtcher retinopathy can be wrongly suspected. Moreover, the ocular involvement with multiple CWS, and retinal whitening may also lead to a misdiagnosis of viral retinitis and not to a Purtscher-like retinopathy especially when presenting as an initial manifestation of SLE. This is a sight threatening condition if not immediately appropriately treated. Similarly, missing a diagnosis of GCA with presence of isolated CWS can lead to devastating visual outcomes. We provided insights into distinct clinical features and specific findings on multimodal imaging in the outline above. OCTA may be useful by demonstrating a decrease in retinal vessel density as a marker of SLE retinopathy. Moreover, the delayed choroidal perfusion on FA can be a marker of GCA.

The relative rarity of some diseases, like ocular amyloidosis that can present as a choroidal amyloid angiopathy mimicking an intermediate/ posterior uveitis makes the diagnosis even more challenging.

Drug and vaccine related etiologies of uveitis have been increasingly important to consider. Indeed, growing data has implicated targeted cancer therapies (i.e., ICIs or MEKi), intravitreal injections and vaccines with intraocular inflammation. Macular edema or a serous retinal detachment can be a VKH-like disease secondary to ICIs or a MEK inhibitors retinopathy. The recent reports of posterior uveitis cases (including MEWDS, APMPEE, ampiginous choroiditis, VKH, herpetic disease and associated uveitis) after COVID-19 vaccines are particularly worrisome, especially when considering future COVID-19 vaccinations.

A high index of suspicion must be maintained for masqueraders of posterior uveitis, which can include serious inflammatory, infectious, and neoplastic disorders. This study highlights the overlapping features of posterior uveitis and retinal conditions and mimickers of posterior uveitis. Careful review of past uveitis history, current medications and recent vaccinations, along with detailed examination looking for signs of past or present inflammation and distinct findings on multimodal imaging may all be required to make the correct diagnosis.

Making the correct diagnosis for posterior uveitis and its masqueraders can be challenging even for the experienced uveitis or retina specialist, and one must maintain a broad differential initially to identify rare entities. This review provides insights into distinct clinical features and specific findings on multimodal imaging. We hope it will aid the general ophthalmologist or retina specialist to narrow the diagnosis to the correct one, perhaps preventing unnecessary vision loss in a patient who presents with an atypical clinical picture.

## Method of literature search

Databases and registries that were searched included Pubmed/Medline, EBSCO, and Cochrane Library to keywords and subject headings defined in Supplementary Table 1. Briefly, the search terms used were: Posterior uveitis OR Panuveitis OR inflammatory Uveitis OR Infectious Uveitis OR Neoplastic Uveitis OR Masquerader syndrome OR Uveitis mimickers OR medication induced uveitis OR White-Dot-Syndromes OR Post-Vaccination Uveitis). The search timeframe was not limited by a specific date, but rather by the results of the articles retrieved.

The retrieved articles were initially screened by title and abstract, and articles with the relevant titles were then screened by full text using predefined inclusion and exclusion criteria. Inclusion criteria. The full article was screened in cases where the relevance was unclear from the abstract. Relevant articles were ultimately compiled into a database and removed of duplicates.

Inclusion criteria for articles were: 1) the paper must be written in or available in English and 2) the paper discussed the presentation and management of masquerade syndromes, inflammatory and infectious ocular diseases and uveitis. Exclusion criteria included 1) the paper concerned patients only with other inflammatory ocular diseases and uveitis 2) the paper did not clearly diagnose the patient with masquerade syndromes 3) citations were from grey literature.

### Supplementary Information

**Additional file 1.**

**Additional file 2: Supplemental Table.** Categories of differential diagnoses of posterior uveitis masqueraders.

## Data Availability

Not applicable.
