# Supplementary figures and images for: Challenges in posterior uveitis—tips and tricks for the retina specialist
Source: J Ophthalmic Inflamm Infect. 2023 Aug 17;13:35. doi: 10.1186/s12348-023-00342-5 (PMC10435440; doi:10.1186/s12348-023-00342-5)

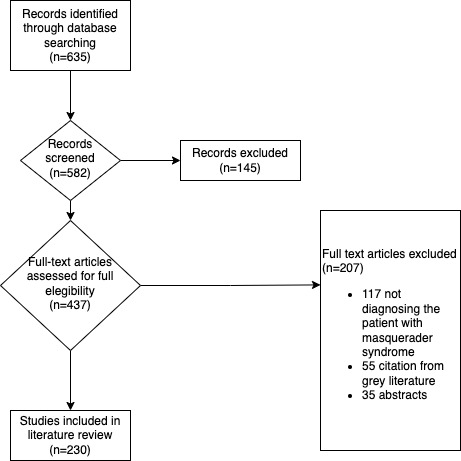

Supplement: Supplementary file 1 — Additional file 1. [file 12348_2023_342_MOESM1_ESM.jpg]
