# Supplementary material for: Challenges in posterior uveitis—tips and tricks for the retina specialist
Source: J Ophthalmic Inflamm Infect. 2023 Aug 17;13:35. doi: 10.1186/s12348-023-00342-5 (PMC10435440; doi:10.1186/s12348-023-00342-5)
Supplement: Supplementary file 2 — Additional file 2: Supplemental Table. Categories of differential diagnoses of posterior uveitis masqueraders. [file 12348_2023_342_MOESM2_ESM.docx]

**Supplemental Table. Categories of differential diagnoses of posterior uveitis masqueraders**

| **Category** | **Etiology** | **Specific overlapping/ mimicking feature of the disease** | **Features (clinical/investigation) help us to differentiate that entity from other diagnoses** |
| --- | --- | --- | --- |
| **Drug related** | Immune check-point inhibitors | VKH-like uveitis | Interview questions by Physician include medications |
|  | MEK inhibitor-associated retinopathy | Retinopathy with central serous retinopathy, sub-foveal retinal detachment, macular edema | Interview questions by Physician include medications |
|  | Anti-TNF-alpha and uveitis | Paradoxical inflammatory effects of anti-TNF-alpha with uveitis, sterile endophthalmitis, andretinal toxicity have been reported under adalimumab.  Uveitis, scleritis with etanercept. | Interview questions by Physician include medications |
|  |  |  |  |
|  | Post-vaccination uveitis (COVID-19) | MEWDS, acute posterior multifocal placoid pigment epitheliopathy, ampiginous choroiditis, exacerbation of VKH | Interview questions by Physician include medications, vaccines |
|  | BCG | bilateral panuveitis, chorioretinitis and/or optic neuritis |  |
|  | tuberculin testing | panuveitis, multifocal choroiditis and VKH disease with serous retinal detachment |  |
|  | MMR | anterior uveitis and panuveitis |  |
|  | influenza | bilateral panuveitis, recurrent panuveitis, acute posterior multifocal placoid pigment epitheliopathy (APMPPE) and reactivation of acute retinal necrosis (ARN) |  |
|  | Others: hepatitis B, HPV and varicella vaccines | uveitis |  |
|  |  |  |  |
| **Neoplastic conditions** | Leukemia, lymphoma | Panuveitis: anterior uveitis, vitritis, white chorioretinal lesions, papillitis and vasculitis. Others manifestations: ischemic bilateral retinal vasculitis and goniosynechae, with associated retinal detachment and a sub-retinal space occupying lesion. | Interview questions by Physician include PMHx, lab work (CBC), Physical exam (swollen lymph nodes ± biopsy, splenomegaly), Imaging (PET scan, MRI), Bone marrow biopsy for systemic lymphoma, lumbar puncture (immunophenotyping) for IOL, ± vitreous biopsy for IOL |
|  | Others: uveal melanoma, retinoblastoma, bilateral diffuse uveal melanocytic proliferation, carcinomas metastatic to the eye, cancer-associated retinopathy (CAR), and melanoma-associated retinopathy (MAR) | Panuveitis, retinal vasculitis, sub-retinal space occupying lesion(s). | Interview questions by Physician include PMHx, Physical exam to rule out carcinoma, melanoma, ± PET scan, CAR syndrome: diagnosis made by a combination of the patient's clinical symptoms, exam findings, diagnosis of systematic cancer, and positive antibodies against retinal proteins; Retinoblastoma: fundus exam; B-scan ultrasound (hyperechoic intraocular mass with hyper-reflective foci and associated posterior shadowing consistent with calcium for retinoblastoma); orbit CT or MRI (retinoblastoma) |
| **Retinal vascular diseases** | GCA | Cotton-wool spots | Physical examination (tenderness temporal arteries), blood test (Erythrocyte sedimentation rate test, C-reactive protein test), BAT |
|  | Ocular ischemic syndrome, CRVO | Retinal vasculitis | carotid Doppler is the first-line test to confirm the diagnosis of carotid artery stenosis |
|  |  |  |  |
| **Idiopathic eye-limited disorder** | CSCR | inflammatory exudative retinal detachment; multifocal choroiditis, such as presumed ocular histoplasmosis syndrome, may be associated with an exudative macular detachment in association with CNV | FA showing one or more fluorescent spots with fluid leakage (in 10%-15%: "classic" smokestack shape), |
|  | Coats disease | Posterior uveitis | The birth history, medical history, and family history are  almost always negative in patients with Coats disease.  Coats : idiopathic retinal telangiectasia and retinal exudation, usually unilateral (95%), mainly  in young males (75%), and possible  exudative retinal detachment.  Slit-lamp biomicroscopy  usually shows normal findings and no cells.  Coats: FA shows early hyperfluorescence of telangiectasia, hypofluorescence of  exudation, and late, mild hyperfluorescence of the  subretinal fluid.  In advanced Coats disease, ultrasonography  shows a linear echo typical of RD. The  subretinal fluid is usually acoustically clear, but may  show few prominent echoes consistent with diffuse  subretinal cholesterolosis. |
|  | Myopic degeneration | MEWDS | Refraction (highly nearsighted eye), retinal imaging (FAF, SD-OCT, ICG) to rule out MEWDS) |
|  | drusen | PIC | OCT |
|  | RRD (Schwartz-Matsuo syndrome when increased intraocular pressure) | anterior chamber inflammation and exhibit features usually seen in uveitic serous detachments, specifically diffuse choroidal thickening, choroidal detachment, and/or white blood cells as well as fibrin in anterior chamber. Other clinical features:  panuveitis with anterior uveitis and hypotony | Fundus examination, B-scan ultrasound (to rule out a retinal tear) |
|  |  |  |  |
| **Hereditary retinal disease and vitreoretinopathies** | retinitis pigmentosa, macular dystrophy,  TRAPS, autosomal recessive bestrophinopathy | chronic posterior uveitis, chronic retinal vasculitis, macula edema, chorioretinis | Genetic tree, genetic testing |
|  | ADNIV | uveitis and vitreoretinal degeneration. The condition progresses to retinal degeneration, peripheral arterial closure, peripheral retinal neovascularization, tractional retinal detachment and neovascular glaucoma | Genetic testing |
|  | FEVR | retinal traction, peripheral vitreous opacities, and subretinal and intraretinal exudates | Genetic tree, familial fundus examination, genetic testing |
| **Systemic Disorder** | SLE: Purtscher-like lupus retinopathy | Purtscher retinopathy | Presence of ANA might indicate lupus. To confirm, anti dsDNA antibodies, ± skin biopsy, ± kidney biopsy, CBC,  urine analysis, low complement levels |
|  | Amyloidosis:  -Retinal angiopathy (microaneurysms, retinal cotton-wool spots, dot  hemorrhages, telangiectasia, and vascular sheathing, ischemic retinopathy).  -choroidal amyloid angiopathy (ICG showing specific aspect with late hypercyanescent delineation of  the choroidal arterial vasculature) | Any posterior uveitis with vitreous opacities | Tissue biopsy (evidence of characteristic amyloid deposits)  search for plasma cell dyscrasia in AL amyloidosis for memory B cells producing aberrant immunoglobulins or portions of immunoglobulins (Immunofixation electrophoresis of urine or serum), genetic testing to look for specific known mutations in transthyretin that predispose to amyloidosis) |

TNF: tumor necrosis factor, MEK: mitogen-activated protein kinase, VKH: Vogt-Koyanagi-Harada; ON: optic neuritis; COVID-19: Coronavirus disease 2019, BCG: bacille Calmette-Guerin, MMR: Measles, Mumps and Rubella, HPV: human papilloma virus, VEGF: vascular endothelial growth factor, GCA: Giant cell arteritis, CRVO: central retinal venous occlusion, CSCR: central serous chorioretinopathy, RRD: rhegmatogenous retinal detachment, PIC: punctate inner choroidopathy; PMHx: past medical history; SLE: systemic lupus erythematosus; TRAPS: TNF-receptor-associated periodic syndrome, ADNIV: autosomal dominant neovascular inflammatory vitreoretinopathy, FEVR: familial exudative vitreoretinopathy; IOL: intraocular lymphoma, TAB: temporal artery biopsy, FA: Fluorescein angiography, FAF: fundus autofluorescence, ICG: indocyanine green angiography, CNV: choroidal neovascularization, ANAL Antinuclear antibody
